# Clinical outcomes of patients with and without diabetes mellitus after hepatectomy: A systematic review and meta-analysis

**DOI:** 10.1371/journal.pone.0171129

**Published:** 2017-02-09

**Authors:** Qingshan Li, Yue Wang, Tao Ma, Yi Lv, Rongqian Wu

**Affiliations:** Shaanxi Center for Regenerative Medicine and Surgical Engineering, Institute of Advanced Surgical Technology and Engineering, Department of Hepatobiliary Surgery, First Affiliated Hospital, Xi’an Jiaotong University School of Medicine, Xi’an, Shaanxi Province, China; Chang Gung Memorial Hospital Kaohsiung Branch, TAIWAN

## Abstract

**Background:**

Clinical data regarding the influence of diabetes mellitus (DM) on the outcomes of patients undergoing hepatectomy are conflicting. To determine the impact of DM on the clinical outcomes of patients undergoing hepatectomy, we systematically reviewed published studies and carried out a meta-analysis.

**Methods:**

A systematic literature search of Pubmed, Sciencedirect, Web of Science, and Chinese Biomedical Database was conducted from their inception through February 2, 2016. The combined relative risk (RR) or hazard ratio (HR) with 95% confidence intervals (95% CI) was calculated.

**Results:**

A total of 16 observational studies with 15710 subjects were eligible for meta-analysis. The pooled results showed that DM significantly increased the risk of overall postoperative complications (RR 1.34; 95% CI 1.19–1.51; P<0.001), DM-associated complications (RR 1.8; 95% CI 1.29–2.53; P<0.001), liver failure (RR 2.21; 95% CI 1.3–3.76; P = 0.028) and post-operative infections (RR 1.59; 95% CI 1.01–2.5; P = 0.045). In addition, DM was also found to be significantly associated with unfavorable overall survival and disease free survival after liver resection. The pooled HR was 1.63 (95% CI 1.33–1.99; P<0.001) for overall survival and 1.55 (95% CI 1.07–2.25; P = 0.019) for disease free survival.

**Conclusion:**

DM is associated with poor outcomes in patients undergoing hepatectomy. DM should be taken into account cautiously in the management of patients undergoing hepatectomy. Further prospective studies are warranted to explore effective interventions to improve the poor outcomes of diabetic patients undergoing hepatectomy.

## Introduction

Diabetes mellitus (DM) is a common chronic disease that can cause widespread health problems. It is an independent risk factor of perioperative complications and mortality in cardiovascular surgeries[1–3]. However, clinical data regarding the influence of DM on the outcomes of patients undergoing hepatectomy are conflicting[4–7], with some studies showing poorer prognosis in DM patients than non-DM patients after liver resection and others showing no difference. Moreover, DM can lead to concomitant impairment in function in various organs. It remains unclear whether DM increases postoperative morbidity after corrected for possible DM-associated organ dysfunction such as cardiac complications and renal insufficiencies. Given the lack of well-powered studies in diabetic patients undergoing hepatectomy and the conflicting results of individual studies, we conducted the following meta-analysis. Our objective was to summarize and compare existing data on the impact of DM on the clinical outcomes of patients undergoing hepatectomy.

## Methods

This meta-analysis was performed in accordance with Preferred Reporting Items for Systematic Reviews and Meta-Analyses (PRISMA) guidelines[8]. We followed the Meta-analysis of Observational Studies in Epidemiology (MOOSE) consensus in this meta-analysis[9].

### Study strategy

A systematic literature search of Pubmed, Sciencedirect, Web of Science, and Chinese Biomedical Database (CBM) was conducted from their inception through February 2, 2016. We combined search terms for diabetes mellitus (“diabetes” or “hyperglycemia”), hepatectomy (“liver resection”) and outcome (“complication”). No language restrictions were applied. We subsequently searched and evaluated all reference lists of eligible articles to identify additional published articles not indexed in the common databases. Study selection was performed by two of us independently (Yue Wang and Qingshan Li), with disagreement resolved by consensus among all the authors.

### Study eligibility

We included studies that met the following criteria: 1) human studies with participants undergoing a surgical procedure of hepatectomy; 2) they reported the association of DM with perioperative morbidity and mortality (any complication or death occurring within 30 days of operation), overall survival (OS; date of surgery to date of death as a result of any cause), and disease-free survival (DFS; date of surgery to date of first recurrence or death); 3) DM was defined as a fasting plasma glucose level of >7.0 mmol/L (126 mg/dL), or a plasma glucose level of >11.1 mmol/L (200 mg/dL) at 2 h in a 75-g oral glucose tolerance test, or the need for insulin or an oral hypoglycemic drug to control glucose levels; 4) hazard ratio (HR) or relative risk (RR) with its 95% confidence interval (95% CI) could be calculated for any of the outcomes. Only full papers and published studies in the medical literature were included. Abstracts, review articles, editorials, case reports, and letters were excluded. In case of multiple publications from the same institution with identical or overlapping patient cohorts, only the most informative publication was included. If a study met all the inclusion criteria but did not report outcomes of interest, we contacted the corresponding author to obtain these data. In all cases of contact, open-ended questions were used to obtain further details. If we could not obtain this information, the study was excluded from the analysis.

### Data extraction and quality assessment

From each retrieved study, the following data were extracted: the first author name, year of publication, study design, country, number of subjects, the mean age of subjects, sex distribution, cause of surgery, the RRs (or HRs) overall and in each subgroup and the corresponding CI. The retrieved studies and extracted data from each included study were independently assessed by two investigators (Yue Wang and Qingshan Li). Any inconsistencies were resolved through consensus with a third author (Tao Ma) for adjudication. Study quality for cohort studies in this meta-analysis was independently assessed with Newcastle-Ottawa quality assessment scale (NOS) as recommended by the Cochrane Non-Randomized Studies Methods Working Group. This instrument uses a “star system” to evaluate data quality. The system criteria included three broad perspectives: the selection (four stars), comparability (two stars) and outcome (three stars); the quality scores of studies range from zero (lowest) to nine (highest)[10]. A score of five or greater was considered high quality, whereas scores less than four were considered low quality.

### Definitions

All postoperative complications were reviewed for at least 90 days after surgery. To correct for possible DM-associated organic complications, we divided the postoperative complications into DM-associated complications, including cardiac complications and renal insufficiencies, and other complications, including liver-related complications and postoperative infections. Liver-related complications include ascites, bile leakage, or liver failure.

### Statistical analysis

We calculated the pooled RR with its corresponding 95% CI to estimate the associations between DM and perioperative morbidity and mortality after hepatectomy. HR with its corresponding 95% CI was also used to assess the associations of DM with OS and DFS. In case of significant heterogeneity, random effects models using the DerSimonian and Laird’s method were employed to allow for it, otherwise a fixed-effects model using the Mantel-Haenszel’s method was created. The presence of heterogeneity across individual studies was evaluated by the Q statistic and the I^2^ statistic (I^2^ > 50% indicated evidence of heterogeneity). For additional analyses, meta-analyses were stratified to explore potential sources of heterogeneity. We further performed sensitivity analysis by sequential omission of individual studies or by omitting studies without high quality. In addition, Egger’s linear regression test, Begg’s rank correlation test and contour-enhanced funnel plots were applied in order to assess the potential publication bias[11,12]. STATA version 12.0 Stata Corp, College Station, TX, USA) was used for all statistical analyses. The analysis was conducted independently and in a double blind manner by two investigators (Yue Wang and Tao Ma). A P value<0.05 was considered statistically significant, except where otherwise specified.

## Results

### Study identification and characteristics

**Fig 1:**
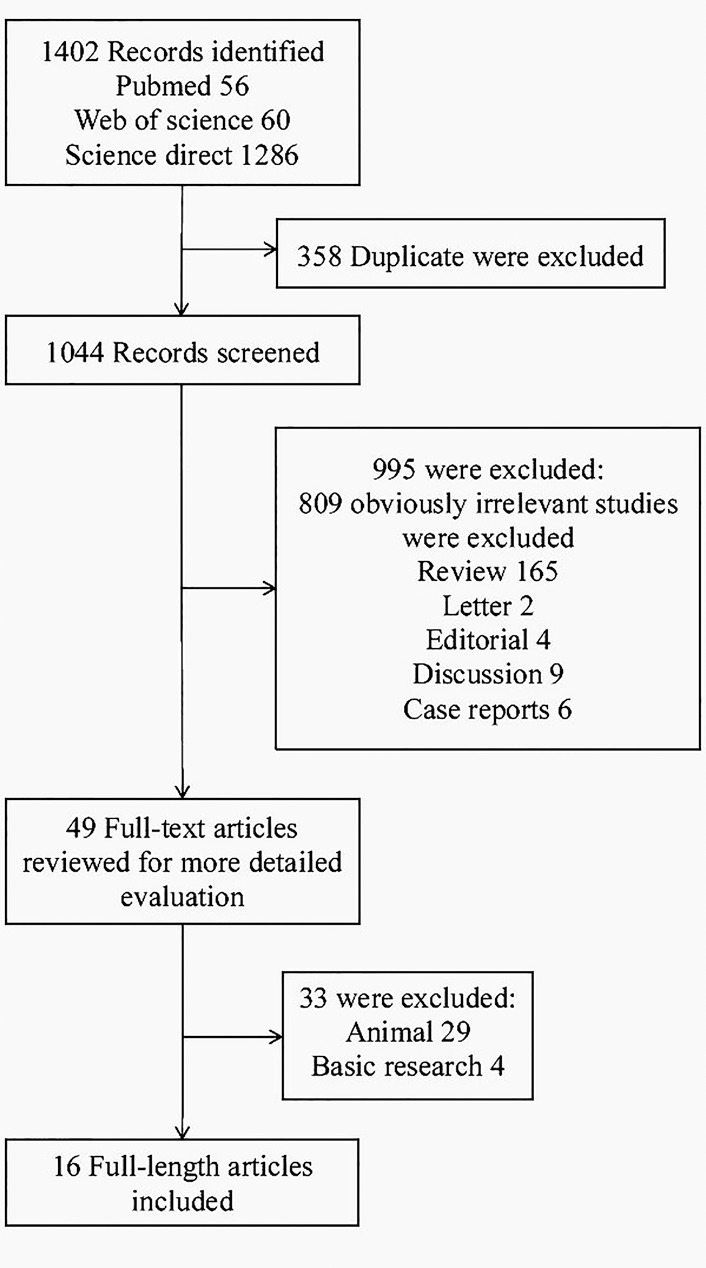
Flow diagram of study selection.

The initial literature search yielded 1402 citations in total. After elimination of duplicate results, 1044 articles were left for screening. 49 were determined to be potentially eligible. Following detailed evaluation which included full text reviews and quality assessment, 33 articles were excluded. The remaining 16 articles corresponding to 15 studies met the inclusion criteria and were included in this meta-analysis[4,7,13–26] (Fig 1).

The main characteristics of all eligible studies are summarized in Table 1 (Table 1). Among these sixteen cohort studies, eleven in Asia, two in North America and three in Europe. A total number of 15710 eligible patients were included. The mean age of subjects ranged from 45.9 to 65.0 years old, and the duration of the studies ranged from 2 to 5.5 years. DM was observed in 28.4% (95%CI 28.0% to 28.8%) patients undergoing hepatectomy. Ten of the fifteen studies with a total of 14410 patients presented data of postoperative complications. Twelve studies with a total of 9044 patients investigated the OS. And DFS was reported in six studies with a total of 1751 patients (Table 1). Assessment of study quality based on Newcastle-Ottawa quality assessment scale (NOS) was shown in Table 1.

**Table 1:** Characteristics of studies included in this meta-analysis.

Study authors	Year	Region	Study design	Disease for hepatectomy	Sample size	Mean age(year)	Gender(male %)	Follow up	Outcome	Quality score
					DM/NDM	DM/NDM	DM/NDM			
Sarah A et al	2002	America	Cohort	Colorectal carcinoma metastases	61/666	NP	NP	24 months	OS; Morbidity; Postoperative morbidity	6
Tsai MS et al	2014	Asia	Cohort	HCC	2962/2962	63.5/62.8	71.8/72.4	NP	Morbidity	8
Huo TI et al	2003	Asia	Cohort	HCC	40/205	65/60	88/85	27 months	Hepatic Decompensation	4
Guckelberger et al	2006	Central Europe	Cohort	Malignancies;Benign tumors;Biliary lesions	75/558	67/54	40/72	NP	Morbidity	4
Amptoulach et al	2015	Northern Europe	Cohort	Colorectal cancer metastases	25/182	NP	NP	37 months	OS; DFS; Morbidity	4
Poon et al	2002	Asia	Cohort	HCC	62/463	60.5/52.4	77/82	54 months	OS; DFS; Morbidity; Postoperative morbidity	6
Yanaga et al	1993	Asia	Cohort	HCC; Cholangiocellular carcinoma;Metastatic liver disease;Hemangioma	49/160	56.7/56.5	90/71	66 months	OS;Morbidity	6
Wang et al	2014	Asia	Cohort	HCC	99/99	54.3/52.5	91/90	31 months	OS;DFS;Postoperative morbidity	7
IKEDA et al	1998	Asia	Cohort	HCC	87/255	59.2/59.8	93/76	42.6 months	Morbidity;OS; DFS	7
Kuroda et al	2011	Asia	Cohort	HCC	66/84	NP	NP	34.2 months	OS	5
Neal et al	2012	Western Europe	Cohort	Benign and Metastatic diseases	7/96	NP	NP	NP	Morbidity;Perioperative Mortality	4
Ting et al	2012	Asia	Cohort	HCC	117/272	63.5/60.9	70.9/72.4	60 months	OS; DFS	6
Huo TI et al	2004	Asia	Cohort	HCC	41/214	64.5/60.2	81.1/88.0	33 months	OS	6
Ou DP et al	2007	Asia	Cohort	HCC	36/374	52.5/46.9	83.3/90.1	58 months	OS	6
Komura T et al	2007	Asia	Cohort	HCC	30/60	62.0/60.6	80/85	60 months	OS; DFS	7
Newhook et al	2016	America	Cohort	Benign and Metastatic diseases	744/4798	NP	NP	NP	Morbidity; OS	5

Abbreviations: DM, diabetes mellitus; NDM, non-diabetes mellitus; OS, overall survival; DFS, disease-free survival; HCC, hepatocellular carcinoma; NP, not reported.

### DM and overall postoperative complications after hepatectomy

**Fig 2:**
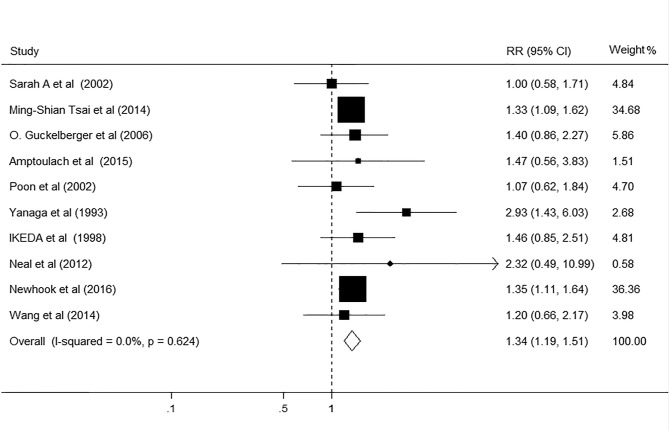
Forest plot on the associations between DM and overall postoperative complications after hepatectomy. DM, diabetes mellitus. The boxes and lines indicate the relative ratios (RRs) and their confidence intervals (CIs) on a log scale for each study. The pooled RR is represented by a diamond. The size of the black squares indicates the relative weight of each estimate.

The relationship between DM and risk of postoperative complications after hepatectomy was evaluated in 10 studies, comprising 14410 participants (Table 1). Among these studies, seven showed no association between DM and overall complications, whereas three showed DM significantly increased risk for postoperative complications after hepatectomy. The pooled results of the meta-analysis revealed that DM significantly increased risk for overall postoperative complications (RR, 1.34; 95% CI, 1.19–1.51; p<0.001; Fig 2), using the fixed effect model (I^2^ = 0.0%, P = 0.539). A 34% relative increase in the risk of postoperative complications in DM patients after hepatectomy was observed in the pooled results. The results of the stratified analysis based on high quality studies showed the association between DM and overall postoperative complications remained robust (RR, 1.33; 95% CI, 1.18–1.51; p<0.001; Table 2). In sensitivity analyses, exclusion of any single study from the analyses did not markedly influence the overall results. The Egger’s test (P = 0.494) and Begg’s test (P = 0.18) suggested there is no significant asymmetry of the funnel plot, indicating the absence of substantial publication bias.

**Table 2:** Stratified analysis of the association between diabetes mellitus and prognosis after hepatectomy.

Endpoint analysed	Studies	Pooled HR or RR	95% CI	Heterogeneity	
				I^2^	
OS	12	1.63	1.33, 1.99	59.0%	
Study quality					
High	11	1.57	1.29, 1.90	54.4%	
Low	1	3.68	1.67, 8.11	NA	
Etiology					
HCC	8	1.59	1.29, 1.96	54.1%	
HCC and others	1	1.63	0.93, 2.86	0.0%	
others	3	1.62	0.68, 3.89	81.3%	
DFS	6	1.55	1.07, 2.25	83.1%	
Study quality					
High	5	1.49	0.99, 2.23	85.6%	
Low	1	2.07	1.11, 3.87	NA	
Etiology					
HCC	5	1.49	0.99, 2.23	85.6%	
HCC and others	0	NA	NA	NA	
others	1	2.07	1.11, 3.87	NA	
Overall complication	10	1.34	1.19, 1.51	0.0%	
Study quality					
High	7	1.33	1.18, 1.51	8.8%	
Low	3	1.46	0.96, 2.22	0.0%	
Etiology					
HCC	4	1.30	1.10, 1.54	0.0%	
HCC and others	2	1.76	1.18, 2.64	64.2%	
others	4	1.32	1.10, 1.57	0.0%	
Hepatic decompensation	7	2.21	1.30, 3.76	57.6%	
Study quality					
High	4	2.36	0.75, 7.42	75.9%	
Low	3	2.00	1.36, 2.93	0.0%	
Etiology					
HCC	3	1.64	0.82, 3.28	28.6%	
HCC and others	2	1.79	1.12, 2.86	0.0%	
others	2	6.5	1.44, 29.28	55.4%	
Infection	6	1.59	1.01, 2.50	71.6%	
Study quality					
High	5	1.52	0.9, 2.57	75.7%	
Low	1	2.05	0.98, 4.29	NA	
Etiology					
HCC	3	1.15	0.85, 1.57	27.2%	
HCC and others	2	1.45	6.35, 0.14	54.1%	
others	1	2.03	0.87, 4.75	NA	

Abbreviations: OS, overall survival; DFS, disease-free survival; CI, confidence intervals; RR, pooled relative risk; HR, pooled hazard risk; NA, not applicable because only one study.

### DM and DM-associated complications after hepatectomy

**Fig 3:**
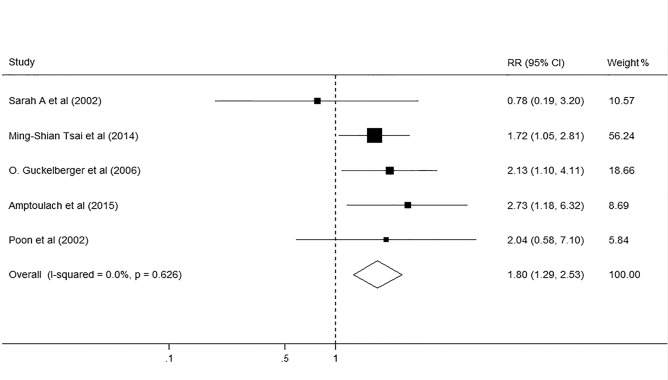
Forest plot on the associations between DM and DM-associated complications after hepatectomy. DM, diabetes mellitus. The boxes and lines indicate the relative ratios (RRs) and their confidence intervals (CIs) on a log scale for each study. The pooled RR is represented by a diamond. The size of the black squares indicates the relative weight of each estimate.

People with DM are often associated with poor circulation and impaired immune responses, which *per se* can lead to concomitant impairment in function in various organs. To correct for possible DM-associated organic complications, we divided the postoperative complications into DM-associated complications, including cardiac complications and renal insufficiencies, and other complications, including liver-related complications and postoperative infections. Among the five studies reporting DM-associated complications, three showed DM increased the incidence of DM-associated complications after hepatectomy, while the other two did not. The summarized RR of this meta-analysis was 1.8 (95% CI, 1.29–2.53; p<0.001; I^2^ = 0.0%, p = 0.626 Fig 3.). The sensitivity analysis identified that the overall results remained stable by excluding any single study from the analysis. No publication bias was found in the included studies (Begg’s test p = 0.624; Egger’s test p = 0.721).

### DM and liver-related complications after hepatectomy

**Fig 4:**
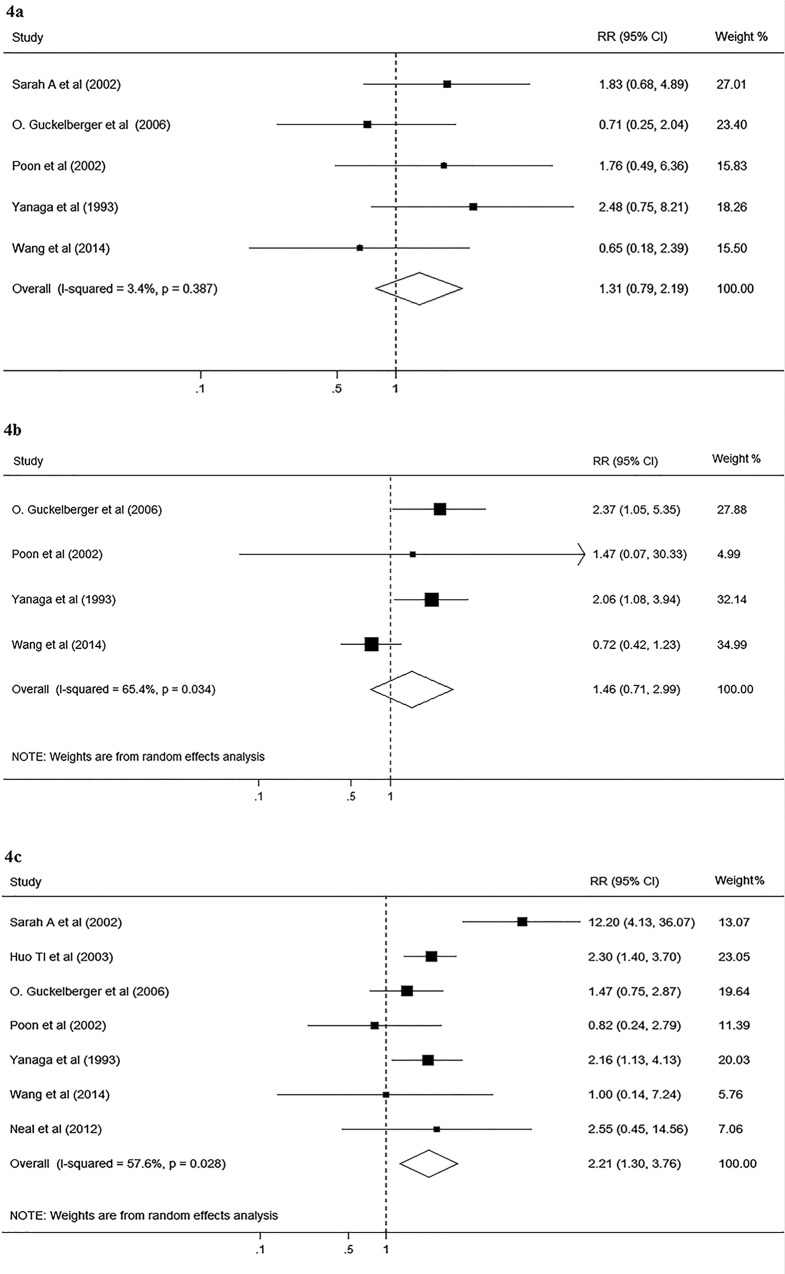
Forest plot on the associations between DM and liver-related complications after hepatectomy. 4a, Forest plot on the associations between DM and bile leakage after hepatectomy. 4b, Forest plot on the associations between DM and ascites after hepatectomy. 4c, Forest plot on the associations between DM and liver decompensation after hepatectomy. DM, diabetes mellitus. The boxes and lines indicate the relative ratios (RRs) and their confidence intervals (CIs) on a log scale for each study. The pooled RR is represented by a diamond. The size of the black squares indicates the relative weight of each estimate.

Eight studies with a total of 2847 participants reported data on the effect of DM on liver-related complications after hepatectomy. Of these studies, one investigated the effect of DM on overall liver-related complications, seven on liver failure, five on bile leakage, and four on ascites. The pooled results showed that DM had no significant effect on bile leakage (RR, 1.31; 95% CI, 0.79–2.19; I^2^ = 3.4%, p = 0.387; Fig 4A) and ascites (RR, 1.46; 95% CI, 0.71–2.99; I^2^ = 65.4%, p = 0.034; Fig 4B). However, significant association was found between DM and liver failure (RR, 2.21; 95% CI, 1.3–3.76; I^2^ = 57.6%, p = 0.028; Fig 4C). The result of stratified analysis showed significant association between DM and liver failure in low quality stratification (RR, 2.00; 95% CI, 1.36–2.93).

### DM and postoperative infections after hepatectomy

**Fig 5:**
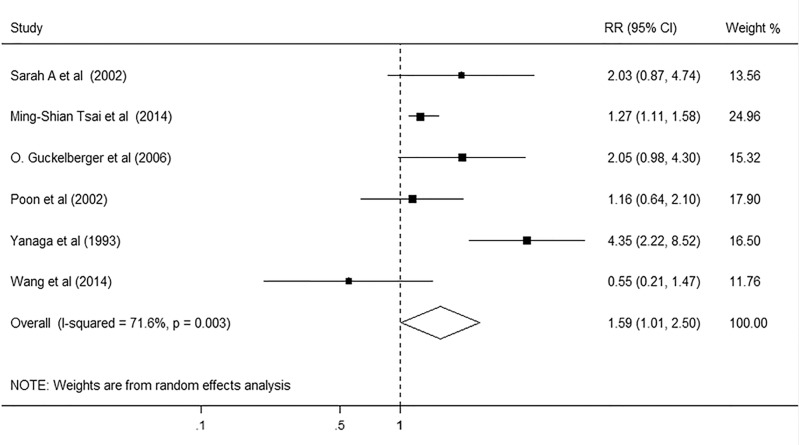
Forest plot on the associations between DM and postoperative infections after hepatectomy. DM, diabetes mellitus. The boxes and lines indicate the relative ratios (RRs) and their confidence intervals (CIs) on a log scale for each study. The pooled RR is represented by a diamond. The size of the black squares indicates the relative weight of each estimate.

We subsequently evaluated the association between DM and the risk for postoperative infections in six studies, comprising 11063 participants. Among these studies, four studies revealed that DM had no significant relationship with the risk for postoperative infections, whereas two studies showed that DM patients had significantly higher risk for postoperative infections. The pooled results of the meta-analysis revealed that DM patients conferred a summarized RR of 1.59 (95% CI, 1.01–2.5; p = 0.045; Fig 5) as compared with non-DM patients, using the random effect model. Although significant heterogeneity was detected across these studies (I^2^ = 71.6%, p = 0.003), the sensitivity analysis identified little influence on the stability of the results after excluding any single study from the analysis. And we did not observe any evidence of publication bias in the included studies (Begg’s test p = 0.851; Egger’s test p = 0.516).

### DM and survival after hepatectomy

**Fig 6:**
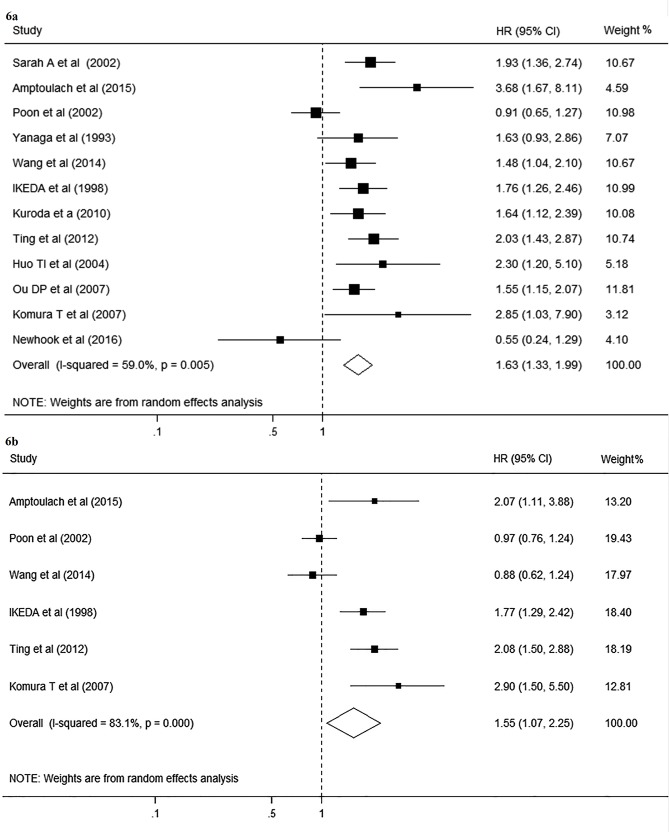
Forest plot on the associations between DM and survival after hepatectomy. 6a, Forest plot on the associations between DM and overall survival after hepatectomy. 6b, Forest plot on the associations between DM and disease-free survival after hepatectomy. DM, diabetes mellitus. The boxes and lines indicate the hazard ratios (HRs) and their confidence intervals (CIs) on a log scale for each study. The pooled HR is represented by a diamond. The size of the black squares indicates the relative weight of each estimate.

**Fig 7:**
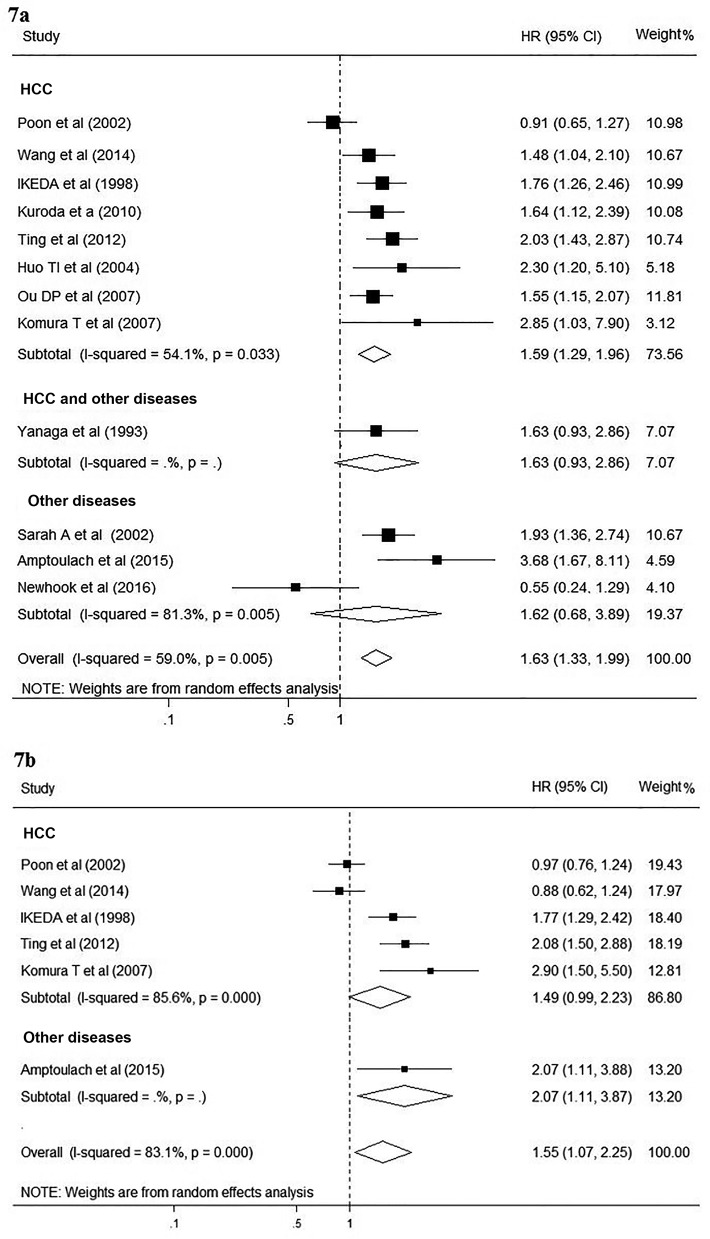
Forest plot on the associations between DM and survival in patients with HCC after hepatectomy. 7a, Forest plot on the associations between DM and overall survival in patients with HCC after hepatectomy. 7b, Forest plot on the associations between DM and disease-free survival in patients with HCC after hepatectomy. DM, diabetes mellitus. The boxes and lines indicate the hazard ratios (HRs) and their confidence intervals (CIs) on a log scale for each study. The pooled HR is represented by a diamond. The size of the black squares indicates the relative weight of each estimate.

Significant heterogeneity was detected in twelve studies about OS (I^2^ = 59.0%; p = 0.005), therefore the random effect model was chosen for the analysis. The pooled HR for OS was 1.63 (95% CI, 1.33–1.99; p<0.001; Fig 6A). The results were not altered when stratified analysis was performed based on study quality. However, when the subgroup analysis was conducted based on the etiology, significant association between DM and poor overall survival was only found in patients with HCC (HR 1.59; 95% CI, 1.29–1.96; Fig 7A). Sensitivity analysis by sequential omission of individual studies did not influence the significance of combined HR estimate. No evidence of publication bias was detected (Begg’s test p = 0.49; Egger’s test p = 0.56).

Similarly, heterogeneity between studies was also significant in six studies about DFS (I^2^ = 83.1%; p<0.001), thus random effect model was used to pool the results. When compared with non-DM patients, DM patients had a significantly lower DFS after liver resection (HR, 1.55; 95% CI, 1.07–2.25; p = 0.019; Fig 6B). However, the subgroup analysis showed significant inconsistency in the direction of effects when studies were confined to HCC patients only (HR, 1.49; 95% CI, 0.99–2.23; Fig 7B). The sensitivity analysis by removing each study one at a time showed the pooled HRs remained stable, indicating that the results were not influenced by any single study. Moreover, no publication bias was detected (Begg’s test p = 0.348; Egger’s test p = 0.209).

## Discussion

DM is generally considered to be an independent risk factor in surgical procedures[27]. However, the prognostic value of DM in hepatectomy remained controversial. Individual studies assessing the effects of DM in patients undergoing hepatectomy had small enrollments and yielded conflicting results[4,7,13,18]. Our meta-analysis of 15 observational studies with a total of 15710 patients addresses this discrepancy by revealing a significant relationship between DM and poor outcomes of patients undergoing hepatectomy. We found sufficient evidence of an increase in the risk of adverse events including overall postoperative complications, liver failure, and infection in diabetic patients undergoing hepatectomy. In addition, DM was found to be significantly associated with unfavorable overall survival and disease free survival after liver resection. Hence, DM should be taken into account cautiously in the management of patients undergoing hepatectomy. Follow-up for such patients should include meticulous glycemic control.

Our findings extend the results from a previous meta-analysis[28]. In their meta-analysis, Wang et al. reported that DM is independently associated with both poorer overall survival (pooled HR 1.55, 95% CI, 1.27–1.91) and poorer disease-free survival (pooled HR 2.15, 95% CI, 1.75–2.63) in HCC patients. However, their study was confined to HCC patients only and they did not assess the association between DM and short-term outcomes. To the best of our knowledge, our current meta-analysis is the first assessment of the literatures regarding DM and the clinical outcomes after liver resection. The strengths of this meta-analysis are the comprehensive strategy that allowed the pooling of almost 16 thousand patients undergoing hepatectomy. In order to investigate the presence of heterogeneity across individual studies, a series of pre-specified sensitivity and subgroup analyses were performed. In case of substantial heterogeneity, the random-effects models were employed to provide the most conservative estimates of the data. This resulted in relatively narrow CIs for pooled proportions of all outcomes. Although we found significant heterogeneities among studies when investigating associations between DM with survival, hepatic decompensation and postoperative infection, the subgroup analyses showed that the primary disease might be a key reason for these heterogeneities. Even so, some of these heterogeneities remained unexplained. The unexplained heterogeneity is likely due to the undefined or unmeasured differences in the study methodology or the differences between the study populations. Furthermore, Funnel plots were constructed in order to assess the potential publication bias. The funnel plot offers a visual sense of the relationship between effect size and precision. The utilization of funnel plots for detecting bias in meta-analysis was discussed in detail by Egger and colleagues [29,30]. Briefly, a symmetric inverted funnel shape arises from a ‘well-behaved’ data set, in which publication bias is unlikely. An asymmetric funnel, on the other hand, suggests the possibility of either publication bias or a systematic difference between studies of higher and lower precision and leads to doubts over the appropriateness of a simple meta-analysis. By using the funnel plots, we found that there was no publication bias in our meta-analysis.

Several potential limitations should also be considered in interpreting the results from this study. As all studies included in this meta-analysis were retrospective cohort studies, the RRs and HRs obtained in this study might be inherently biased by various factors. Therefore, adequately designed prospective studies with an appropriate multivariate analysis taking into account the classical well-defined prognostic factors for hepatectomy, such as tumor number and distribution, Child-Pugh classification, are needed to get a more precise estimate on the prognostic role of DM in patients receiving liver resection. Moreover, the potential publication bias was also a concern. Although we did not observe apparent publication bias through statistical tests, it was still difficult to completely rule out this problem because there were no sufficient amounts of studies to adequately detect biases. And small studies with negative results tend not to be published.

This meta-analysis also showed that the pooled prevalence of DM was much higher in patients undergoing hepatectomy (28.4%) than the prevalence of DM in the general population (4–9%)[31,32]. Since the majority of patients undergoing hepatectomy included in this meta-analysis was HCC patients. This result confirms recent discoveries that those with DM have a higher risk of developing HCC than the general population[33,34].

In conclusion, this meta-analysis provides the best available evidence for an association between DM and poor prognosis of patients after liver resection. DM not only increased the risk of postoperative complications, but also decreased overall survival and disease-free survival in patients after hepatectomy. Further prospective studies are warranted to substantiate these associations and to explore effective interventions to improve the poor prognosis of diabetic patients undergoing hepatectomy.

## Supporting information

S1 Table The PRISMA checklist. (DOC)

S2 Table The raw data extracted from eligible studies. (DOCX)
