# Supplementary material for: Clinical outcomes of patients with and without diabetes mellitus after hepatectomy: A systematic review and meta-analysis
Source: PLoS One. 2017 Feb 9;12(2):e0171129. doi: 10.1371/journal.pone.0171129 (PMC5300262; doi:10.1371/journal.pone.0171129)
Supplement: S2 Table — (DOCX) [file pone.0171129.s002.docx]

1. **Summary table**

| Sequence | Author | Year | Country | Design | Disease for hepatectomy | n | Diabetes  (n) | DM male  (%） | DM mean age（year） | Control  (n) | NDM male  (%) | NDM mean age | Follow up | Outcome | Quality score |
| --- | --- | --- | --- | --- | --- | --- | --- | --- | --- | --- | --- | --- | --- | --- | --- |
| 1 | Sarah A et al | 2002 | USA | Cohort | Colorectal carcinoma metastases | 727 | 61 | NP | NP | 666 | NP | NP | 24 months | OS; Morbidity; Postoperative morbidity | 6 |
| 2 | Tsai MS et al | 2014 | China  (Tai wan) | Cohort | HCC | 5924 | 2962 | 71.8 | 63.5 | 2962 | 72.4 | 62.8 | NP | Morbidity | 8 |
| 3 | Huo TI et al | 2003 | China (Tai wan) | Cohort | HCC | 239 | 39 | 85.0 | 63.0 | 200 | 88.5 | 60.0 | 32 months | OS | 5 |
| 4 | Huo TI et al | 2003 | China  (Tai wan) | Cohort | HCC | 245 | 40 | 88.0 | 65.0 | 205 | 85.0 | 60.0 | 27 months | Hepatic Decompensation | 5 |
| 5 | Guckelberger et al | 2006 | Germany | Cohort | Malignancies；Benign tumors；Biliary lesions | 633 | 75 | 40.0 | 67.0 | 558 | 72.0 | 54.0 | NP | Morbidity | 4 |
| 6 | Amptoulach et al | 2015 | Sweden | Cohort | Colorectal cancer metastases | 207 | 25 | NP | NP | 182 | NP | NP | 37 months | OS; DFS; Morbidity | 4 |
| 7 | Poon et al | 2002 | China (Hong Kong) | Cohort | HCC | 525 | 62 | 77.0 | 60.5 | 463 | 82.0 | 52.4 | 54 months | OS; DFS; Morbidity; Postoperative morbidity | 6 |
| 8 | Yanaga et al | 1993 | Japan | Cohort | HCC; Cholangiocellular carcinoma；Metastatic liver disease；Hemangioma | 209 | 49 | 90.0 | 56.7 | 160 | 71.0 | 56.5 | 5.5years | OS; Morbidity | 6 |
| 10 | Wang et al | 2014 | China | Cohort | HCC | 198 | 99 | 91.0 | 54.3 | 99 | 90.0 | 52.5 | 31 months | OS；DFS；Postoperative morbidity | 7 |
| 11 | IKEDA et al | 1998 | Japan | Cohort | HCC | 342 | 87 | 93.0 | 59.2 | 255 | 76.0 | 59.8 | 42.6 months | Morbidity；OS;DFS | 7 |
| 12 | Kuroda et al | 2011 | Japan | Cohort | HCC | 150 | 66 | NP | NP | 84 | NP | NP | 34.2 months | OS | 5 |
| 13 | Neal et al | 2012 | England | Cohort | Benign and Metastatic diseases | 103 | 7 | NP | NP | 96 | NP | NP | NP | Morbidity；Perioperative Mortality | 4 |
| 14 | Ting et al | 2012 | China  (Tai wan) | Cohort | Cirhosis；HCC | 389 | 117 | 70.9 | 63.5 | 272 | 72.4 | 60.9 | 5 years | OS;DFS | 6 |
| 15 | Huo TI et al | 2004 | China  (Tai wan) | Cohort | HCC | 255 | 41 | 81.1 | 64.5 | 214 | 88.0 | 60.2 | 33 months | OS | 6 |
| 16 | Ou DP et al | 2007 | China | Cohort | HCC | 410 | 36 | 83.3 | 52.5 | 374 | 90.1 | 46.9 | 58 months | OS | 6 |
| 17 | Komura T et al | 2007 | Japan | Cohort | HCC | 90 | 30 | 80.0 | 62.0 | 60 | 85.0 | 60.6 | 5 years | OS, DFS | 7 |
| 18 | Newhook et al | 2016 | USA | Cohort | Benign and Metastatic diseases | 5,542 | 744 | NP | NP | 4798 | NP | NP | NP | Morbidity,OS | 5 |

1. **The association between DM and Disease free survival**

| sequence | author name | year | HR | L | U | country | race | quality score | quality subgroup |
| --- | --- | --- | --- | --- | --- | --- | --- | --- | --- |
| 6 | Amptoulach | 2015 | 2.073 | 1.109 | 3.875 | Sweden | 2 | 4 | 3 |
| 7 | Poon | 2002 | 0.97 | 0.76 | 1.24 | China (Hong Kong) | 1 | 6 | 4 |
| 10 | Wang | 2014 | 0.878 | 0.624 | 1.237 | china | 1 | 7 | 4 |
| 11 | IKEDA | 1998 | 1.77 | 1.29 | 2.42 | Japan | 1 | 7 | 4 |
| 14 | Ting | 2012 | 2.076 | 1.495 | 2.882 | China（Taiwan） | 1 | 6 | 4 |
| 17 | Komura T | 2007 | 2.9 | 1.5 | 5.5 | Japan | 1 | 7 | 4 |

**C. The association between DM and Overall survival**

| Sequence | Author name | Year | HR | L | U | Country |
| --- | --- | --- | --- | --- | --- | --- |
| 1 | Sarah A et al | 2002 | 1.93 | 1.36 | 2.74 | USA |
| 3 | Teh-Ia Huo | 2003 | 1.71 | 1.01 | 2.93 | China（Taiwan） |
| 6 | Amptoulach | 2015 | 3.682 | 1.672 | 8.109 | Sweden |
| 7 | Poon | 2002 | 0.91 | 0.65 | 1.27 | China (Hong Kong) |
| 8 | Yanaga | 1993 | 1.63 | 0.93 | 2.86 | Japan |
| 10 | Wang | 2014 | 1.482 | 1.044 | 2.104 | China |
| 11 | IKEDA | 1998 | 1.76 | 1.26 | 2.46 | Japan |
| 12 | Tashiro | 2010 | 1.637 | 1.117 | 2.392 | Japan |
| 14 | Ting | 2012 | 2.03 | 1.435 | 2.871 | China（Taiwan） |
| 15 | Teh-Ia Huo | 2004 | 2.3 | 1.2 | 5.1 | China |
| 16 | Ou DP | 2007 | 1.55 | 1.15 | 2.07 | China |
| 17 | Komura T | 2007 | 2.85 | 1.03 | 7.9 | Japan |
| 18 | Newhook | 2016 | 0.552 | 0.235 | 1.295 | USA |

1. **The association between DM and Overall Complication**

| Sequence | Author name | Year | RR | L | U | Country |
| --- | --- | --- | --- | --- | --- | --- |
| 1 | Sarah A et al | 2002 | 1 | 0.585 | 1.711 | USA |
| 2 | Ming-Shian Tsai et al | 2014 | 1.328 | 1.087 | 1.623 | China （Tai wan） |
| 5 | O. Guckelberger et al | 2006 | 1.396 | 0.857 | 2.272 | Germany |
| 6 | Amptoulach et al | 2015 | 1.467 | 0.562 | 3.83 | Sweden |
| 7 | Poon et al | 2002 | 1.069 | 0.62 | 1.842 | China (Hong Kong) |
| 8 | Yanaga et al | 1993 | 2.933 | 1.426 | 6.032 | Japan |
| 10 | Wang et al | 2014 | 1.2 | 0.664 | 2.168 | China |
| 11 | IKEDA et al | 1998 | 1.463 | 0.854 | 2.506 | Japan |
| 13 | Neal et al | 2012 | 2.32 | 0.49 | 10.99 | England |
| 18 | Newhook | 2016 | 1.347 | 1.108 | 1.639 | USA |

1. **The association between DM and Acites**

| Sequence | Author name | Year | DM(n1) | x1 | NDM(n2) | x2 | Country |
| --- | --- | --- | --- | --- | --- | --- | --- |
| 5 | O. Guckelberger et al | 2006 | 75 | 7 | 558 | 22 | Germany |
| 7 | Poon | 2002 | 62 | 0 | 463 | 2 | China (Hong Kong) |
| 8 | Yanaga et al | 1993 | 49 | 12 | 160 | 19 | Japan |
| 10 | Wang et al | 2014 | 99 | 18 | 99 | 25 | China |

1. **The association between DM and Bile leakage**

| Sequence | Author name | Year | DM(n1) | x1 | NDM(n2) | x2 | RR | L | U | Country |
| --- | --- | --- | --- | --- | --- | --- | --- | --- | --- | --- |
| 1 | Sarah A et al | 2002 | 61 | 5 | 666 | 31 | 1.829 | 0.684 | 4.889 | USA |
| 5 | O. Guckelberger et al | 2006 | 75 | 4 | 558 | 41 | 0.71 | 0.247 | 2.043 | Germany |
| 7 | Poon et al | 2002 | 62 | 3 | 463 | 13 | 1.76 | 0.487 | 6.358 | China (Hong Kong) |
| 8 | Yanaga et al | 1993 | 49 | 5 | 160 | 7 | 2.484 | 0.751 | 8.21 | Japan |
| 10 | Wang et al | 2014 | 99 | 4 | 99 | 6 | 0.653 | 0.178 | 2.388 | China |

1. **The association between DM and Hepatic decompensation**

| Sequence | Author name | Year | RR | L | U | Country |
| --- | --- | --- | --- | --- | --- | --- |
| 1 | Sarah A et al | 2002 | 12.204 | 4.129 | 36.071 | USA |
| 4 | Teh-Ia Huo et al | 2003 | 2.3 | 1.4 | 3.7 | china (Tai wan) |
| 5 | O. Guckelberger et al | 2006 | 1.47 | 0.752 | 2.873 | Germany |
| 7 | Poon et al | 2002 | 0.821 | 0.242 | 2.791 | China (Hong Kong) |
| 8 | Yanaga et al | 1993 | 2.158 | 1.128 | 4.129 | Japan |
| 10 | Wang et al | 2014 | 1 | 0.138 | 7.243 | China |
| 13 | Neal et al | 2012 | 2.55 | 0.45 | 14.56 | England |

1. The association between DM and Infection

| Sequence | Author name | Year | DM(n1) | x1 | NDM(n2) | x2 | RR | L | U | Country |
| --- | --- | --- | --- | --- | --- | --- | --- | --- | --- | --- |
| 1 | Sarah A et al | 2002 | 61 | 7 | 666 | 40 | 2.029 | 0.867 | 4.745 | USA |
| 2 | Ming-Shian Tsai et al | 2014 | 2962 | 189 | 2962 | 151 | 1.269 | 1.108 | 1.581 | China (Tai wan) |
| 5 | O. Guckelberger et al | 2006 | 75 | 10 | 558 | 39 | 2.047 | 0.976 | 4.296 | Germany |
| 7 | Poon et al | 2002 | 62 | 17 | 463 | 114 | 1.157 | 0.637 | 2.1 | China (Hong Kong) |
| 8 | Yanaga et al | 1993 | 49 | 29 | 160 | 40 | 4.35 | 2.22 | 8.524 | Japan |
| 10 | Wang et al | 2014 | 99 | 7 | 99 | 12 | 0.552 | 0.208 | 1.466 | China |

1. **The association between DM and DM-related complication**

| Sequence | Author name | Year | DM(n1) | x1 | NDM(n2) | x2 | Country |
| --- | --- | --- | --- | --- | --- | --- | --- |
| 1 | Sarah A et al | 2002 | 61 | 2 | 666 | 28 | USA |
| 2 | Ming-Shian Tsai et al | 2014 | 2962 | 43 | 2962 | 25 | China (Tai wan) |
| 5 | O. Guckelberger et al | 2006 | 75 | 10 | 558 | 35 | Germany |
| 6 | Amptoulach et al | 2015 | 25 | 6 | 182 | 16 | Sweden |
| 7 | Poon et al | 2002 | 62 | 3 | 463 | 11 | China (Hong Kong) |
| 8 | Yanaga et al | 1993 | 49 | 0 | 160 | 0 | Japan |
